# The roles of amensalistic and commensalistic interactions in large ecological network stability

**DOI:** 10.1038/srep29929

**Published:** 2016-07-13

**Authors:** Akihiko Mougi

**Affiliations:** 1 Department of Biological Science, Faculty of Life and Environmental Science, Shimane University, 1060 Nishikawatsu-cho, Matsue 690-8504, Japan

## Abstract

Ecological communities comprise diverse species and their interactions. Notably, ecological and evolutionary studies have revealed that reciprocal interactions such as predator–prey, competition, and mutualism, are key drivers of community dynamics. However, there is an argument that many species interactions are asymmetric, where one species unilaterally affects another species (amensalism or commensalism). This raises the unanswered question of what is the role of unilateral interactions in community dynamics. Here I use a theoretical approach to demonstrate that unilateral interactions greatly enhance community stability. The results suggested that amensalism and commensalism were more stabilizing than symmetrical interactions, such as competition and mutualism, but they were less stabilizing than an asymmetric antagonistic interaction. A mix of unilateral interactions increased stability. Furthermore, in communities with all interaction types, unilateral interactions tended to increase stability. This study suggests that unilateral interactions play a major role in maintaining communities, underlining the need to further investigate their roles in ecosystem dynamics.

In natural ecosystems, a diverse number of species interact and coexist with each other. Species interactions that have reciprocal effects on the interacting partners, such as antagonism (predation, herbivory, or parasitism), competition and mutualism, are the driving forces of population and community dynamics^1^. Recent studies have revealed the important roles of such diverse types of reciprocal interaction in community stability and ecosystem functioning^2^,^3^,^4^,^5^,^6^,^7^,^8^,^9^,^10^,^11^,^12^,^13^,^14^,^15^. However, potentially common interactions in natural communities are not reciprocal but unilateral (one species has a marked effect on only one other species). Nevertheless, it remains unclear how such unilateral interactions (amensalism and commensalism) affect the stability of community dynamics^16^.

Amensalism can be defined as an interaction in which one organism inflicts harm to another organism without receiving any costs or benefits. For example, the movement of large terrestrial vertebrates crushes grass and small terrestrial invertebrates^16^. Similarly, the bread mold *Penicillium* kills certain bacteria by producing penicillin. In contrast, commensalism can be defined as an intraspecific relationship in which one species (the commensal) obtains benefits such as food, shelter, or locomotion from another species (the host) without causing adverse effects. Commensalism often occurs between a larger host and a smaller commensal. The host is unaffected, whereas the commensal can receive great benefits. For example, remoras attach to sharks and other fishes, and various biting lice, fleas, and mosquitoes feed harmlessly on the skin of mammals. There are also many organisms that use trees and hermatypic corals as a habitat.

Many interactions are strongly asymmetric^17^,^18^,^19^,^20^,^21^,^22^ (one interaction strength in a pairwise interaction is strong and another is weak) and, therefore, are almost unilateral instead of reciprocal. Species interactions can be classified by a combination of interaction coefficient signs +, −, or 0^23^. Reciprocal interactions include antagonism, competition, and mutualism are defined as (+, −), (−, −), and (+, +), respectively. Unilateral interactions include amensalism and commensalism are defined as (−, 0) and (+, 0), respectively. Antagonism (+, −), such as prey–predator and host–parasite, may be amensalistic (0, −) or commensalistic (+, 0)^17^. Competition (−, −) also may be amensalistic^18^,^19^,^20^. An interaction that is mutualistic (+, +) in one ecological circumstance could be commensalistic in another circumstance^21^,^22^,^24^. The inherent diversity of unilateral interactions^25^ suggests that elucidating the role of unilateral interactions in community dynamics leads to an understanding of whole communities rather than parts of a community^16^.

The diversity of interaction signs is related to the asymmetry of interaction strengths. The interaction compass^26^ shows that interaction types classified by sign combinations transition by changing the interaction strengths. For example, if *a*_ij_ ≈ *a*_ji_ > 0 (where *a*_ij_ is the interaction coefficient), the interaction between species *i* and species *j* is mutualistic (+, +) and symmetric. If *a*_ji_ decreases so that *a*_ij_ > *a*_ji_ > 0, the interaction is still mutualistic but is also asymmetric (+, +). When *a*_ji_ = 0 (one species diminishes its resource supply to other species), the interaction is commensalistic (+, 0). When *a*_ji_ < 0 (one species overexploits the other species), the interaction changes to antagonism or parasitism (+, −). If *a*_ij_ ≈ *a*_ji_ < 0, the interaction is competition (*−*, −). Similarly, it can be highly asymmetric and shift to amensalism (0, −) if one species is the superior competitor. If *a*_ij_ > 0 > *a*_ji_, the interaction is antagonism (+, −). It can be symmetric or asymmetric. For example, intraguild predation (+, −) may shift to mutual predation (*−*, −) if the predator-prey interaction is bi-directional. Predator-prey interaction (+, −) may be highly asymmetric and shift to amensalism (0, −) if the prey is nutritionally poor. Parasitism (+, −) may be also highly asymmetric and shift to commensalism (+, 0) if the parasite uses the host as a shelter.

Interaction networks in natural communities are comprised of such diverse interaction sign types and strengths. Unilateral interactions are intermediate cases when the effect of one species on another transitions from a positive effect to a negative effect and vice versa. In this context, revealing the effects of unilateral interactions in community dynamics facilitates our understanding of the roles of both interaction signs and strengths that are the key elements of natural communities.

A community dynamics model is presented in which reciprocal and unilateral interactions are combined to examine the role of unilateral interactions in community stability. Reciprocal interactions include antagonism, competition, and mutualism, whereas unilateral interactions include amensalism and commensalism. By controlling the asymmetry of interaction strengths in communities with a single interaction type and the composition of unilateral interactions within a whole community with all interaction types, I reveal the effects of unilateral interactions on community stability following May’s approach^1^ (Methods).

## Results

Consider a community where *N* species interact with each other with a probability *C* (connectance) through reciprocal interactions of antagonism, competition, or mutualism and through unilateral interactions of amensalism or commensalism. The proportion of unilateral interactions within a community is defined as *p*_u_ (i.e., the proportion of reciprocal interactions is 1 − *p*_u_). The population dynamics of *N* species and the effect of unilateral interactions on the stability of population dynamics by systematically changing *p*_u_ were evaluated (see Methods).

**Figure 1:**
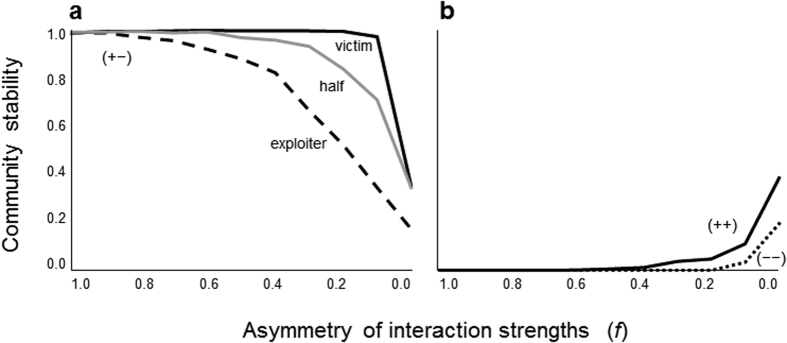
Effects of interaction strength asymmetry on stability of communities with reciprocal interactions (*p*_u_ = 0). (**a**) Antagonistic community, (**b**) Competitive or mutualistic community. Parameter values are *N* *=* 50, *C* *=* 0.2, *s* *=* 4.0, and *σ* *=* 0.5. The interaction strength asymmetry is controlled by asymmetry parameter *f* (see Methods). As *f* decreases, the asymmetry of interaction strengths increases (*f* = 0 is perfect asymmetry). In antagonistic community, the relative strengths of interaction coefficients of victims, exploiters or half of victims and exploiters are changed by multiplying the asymmetry parameter *f* by one interaction coefficient in each interaction pair.

Consider communities with only reciprocal interactions (*p*_u_ = 0). Congruent with a previous study^27^, communities with antagonism are more stable than those with mutualism or competition (Fig. 1). Here I introduce a parameter *f* that controls the asymmetry of interaction strengths (Methods). Controlling the asymmetry of interaction strengths demonstrates the effects of unilateral interactions on community stability (Fig. 1). In antagonistic communities, increasing the asymmetry of interaction strengths decreases stability. Extreme asymmetry leads to communities with unilateral interactions (amensalism and/or commensalism), resulting in less stability than communities with reciprocal interactions. In contrast, increasing the asymmetry of interaction strengths in competitive and mutualistic communities increases stability. These simulations are supported by a mathematical analysis (SI text). The results suggest that unilateral interactions have a more stabilizing effect than symmetrical reciprocal interactions such as competition and mutualism, but more destabilizing effects than asymmetric reciprocal interactions such as predator-prey or host-parasite interactions.

**Figure 2:**
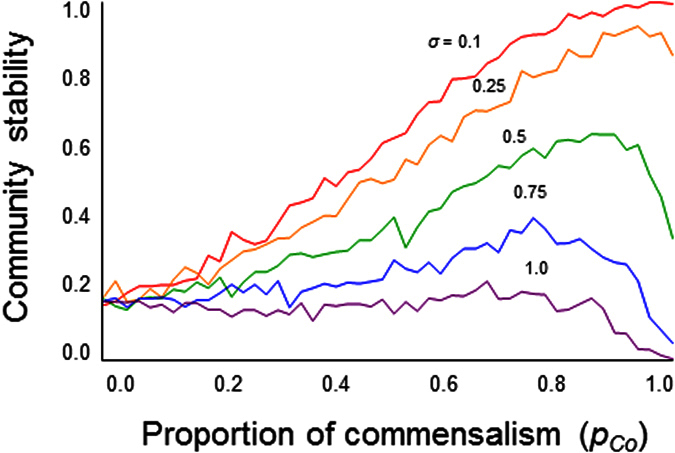
Stability of communities with unilateral interactions (*p*_u_ = 1) with varying proportion of commensalism *p*_Co_. Colors indicate different values of *σ*, the standard deviation of normal distribution n(0, *σ*^2^) from which all parameter values were randomly chosen (Methods). Parameter values are *N* = 50, *C* = 0.2 and *s* = 4.0.

Consider an extreme case where all species interactions are asymmetrical. The communities are then only comprised of unilateral interactions (*p*_u_ = 1). Communities with only unilateral interactions (amensalism and commensalism) tend to need both unilateral interaction types for higher stability (Fig. 2). Communities skewed to either unilateral interaction type are unstable, whereas those with a moderate mix of amensalism and commensalism tend to be more stable. This unimodal pattern of stability is observed in intermediate levels of *σ* (that represents variation of parameters) (Fig. 2). A mathematical analysis shows that this unimodal pattern does not appear without parameter variations (SI text). Furthermore, the overall unimodal pattern observed remains qualitatively unchanged over a wide range of *N* and *C* (Fig. S1), regardless of the distribution from which the parameters are chosen (Fig. S2). This result suggests that mixing of unilateral interactions have a tendency to enhance community stability when all parameters have moderate variations.

**Figure 3:**
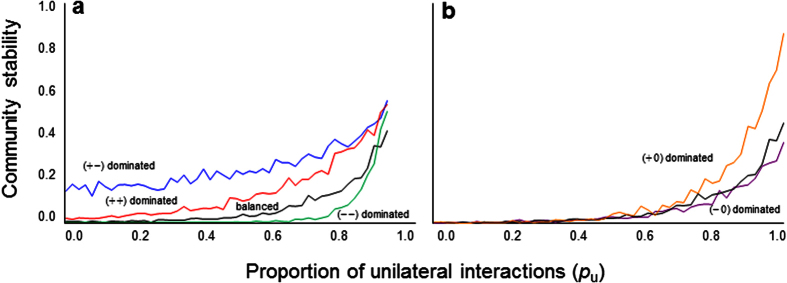
Stability of hybrid communities with reciprocal and unilateral interactions with variable proportions of unilateral interactions *p*_u_. (**a**) Effects of different community composition of the reciprocal interactions, balanced interactions, *p*_*a*_ dominated, *p*_*m*_ dominated, and *p*_*c*_ dominated. Black, blue, red, and green dots indicate different proportions of reciprocal interactions, (*p*_*a*_, *p*_*m*_, *p*_*c*_) = (1/3, 1/3, 1/3), (0.7, 0.15, 0.15), (0.15, 0.7, 0.15) and (0.15, 0.15, 0.7), respectively. It was assumed that *p*_*Co*_ = *p*_*Am*_. (b) Effects of different community composition of the unilateral interactions, balanced interactions, *p*_*Am*_ dominated, and *p*_*Co*_ dominated. Black, purple, and orange dots indicate different unilateral interaction proportions, *p*_*Co*_ = 0.5, 0.1, and 0.9, respectively. It was assumed that *p*_*a*_ = *p*_*m*_ = *p*_*c*_. Parameter values are *N* = 50, *C* = 0.2, *s* = 4.0 and *σ* = 0.3.

Do reciprocal interactions or unilateral interactions result in a more stable community? To answer this question, first consider a scenario in which the proportions of all interaction types are the same. In this case, unilateral interaction communities are more stable than reciprocal interaction communities (*p*_u_ = 0 and *p*_u_ = 1, Fig. 3). This simulation result is supported by a mathematical analysis (SI text). In addition, the result was upheld regardless of the proportions of each interaction type (Fig. 3). Unilateral interactions also compensate for unstable reciprocal interactions (Fig. 3). The stabilization due to unilateral interactions remains qualitatively unchanged over a wide range of *N* and *C* (Fig. S3), regardless of the distributions from which the parameters are chosen (Fig. S4).

What is the key factor that determines stability? The stability due to unilateral interactions is explained by the realized half connectance of reciprocal interactions (SI text). The zero interaction strengths in unilateral interactions have an effect to decrease the connectance by half, resulting in stabilization of communities as predicted by earlier theories^1^,^27^. Although the decrease in the statistical quantities such as the mean, variance, and correlation of the distribution of interaction coefficients is also known to increase stability^27^, these effects on stability are not enough large to explain the effects of *p*_u_ on stability (Figs S5 and S6).

## Discussion

Ecological theory has emphasized the importance of reciprocal species interactions in community dynamics^8^,^27^. In fact, this study also shows that reciprocal interactions such as antagonism, competition, and mutualism largely affect community stability. However, I also revealed that the often overlooked unilateral interactions, amensalism and commensalism, also affect community dynamics in three ways. First, unilateral interactions tend to have a stabilizing effect on community dynamics. Second, communities comprised of only unilateral interactions tend to be more stable than communities with mixed reciprocal interactions, as purely antagonistic communities are more stable than communities with only unilateral interactions. Third, unilateral interactions stabilize otherwise less stable communities with reciprocal interactions. These results suggest that unilateral interactions play a key role in ecosystem dynamics.

Two types of asymmetry in species interactions can have major roles in community stability. First one is the interaction sign asymmetry^27^. The communities with reciprocal interactions with symmetrical signs, mutualism (+, +) or competition (−, −), are inherently more unstable than those with asymmetrical signs, antagonism (+, −). Earlier studies have predicted that the community stability requires any special non-random interaction network structure^22^,^28^,^29^,^30^. However, even without such network structures, mutualism and competition can stabilize population dynamics by coexisting in the community^15^ or by the support of an inherently stable antagonism^27^. A balance of interaction signs within a community may maintain ecological communities.

Second type of asymmetry in species interactions is the interaction strength asymmetry^22^. To achieve community stability, one interaction coefficient must be smaller than the other, and unilateral interactions are an extreme example of this type of asymmetry. This idea was demonstrated in a community with a single interaction type (e.g., mutualism); however, whether the asymmetry of interaction strengths is crucial for the maintenance of communities with other interaction types remains unresolved. The present results support the stabilizing effect of interaction asymmetry in communities with symmetrical interaction signs (mutualistic or competitive communities). In contrast, the interaction strength asymmetry has destabilizing effects in communities with asymmetrical interaction signs (antagonistic communities). If the interaction strengths of interacting partners are asymmetrical, the world is approximately comprised of unilateral interactions with different signs, (+, 0) and (−, 0). Even in such a case, different interaction types stabilize community dynamics by coexisting within a community. Generally, these augments suggest that either interaction signs or strengths is necessary for stabilizing communities.

The importance of asymmetric interaction signs and strengths has been independently shown in previous studies^22^,^27^. Unilateral interactions inevitably involve both types of asymmetry. In a community with unilateral or highly asymmetrical interactions, the asymmetry of interaction strengths and interaction signs (+ and −) leads to a balance in nature. In fact, analysis of field studies suggests that the balance of interaction signs +:− is equal to 1:1 25. In addition, previous theory predicts that unilateral interactions are more common than reciprocal interactions^25^, suggesting that natural ecosystems are stabilized by a balance of different interaction signs or unilateral interactions. Whether the predictions of a local stability analysis captures the natural environment remains open, although the present study strongly suggests the importance of unilateral interactions in community dynamics and ecosystem functioning.

## Methods

Consider a community where *N* species may interact with each other through antagonism, competition, mutualism, or act through amensalism or commensalism. In the model, competition represents direct competition, such as interference competition. If a type I functional response is assumed, the population dynamics of species *i* can be described as follows:


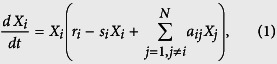


where *X*_*i*_ is the abundance of species *i*, *r*_*i*_ is the intrinsic rate of change in species *i*, *s*_*i*_ is density-dependent self-regulation, and *a*_*ij*_ is the interaction coefficient between species *i* and *j*. A random network^31^ was utilized to only focus on the effects of interaction types on community stability and to respond to limited empirical data in the ecological network, including diverse interaction types. Connectance (C) was defined as the proportion of realized interaction links L in the possible maximum interaction links *L*_*max*_ (=*N*(*N* − 1)/2) of a given network model (*L* = *CL*_*max*_). The interaction coefficient, *a*_*ij*_ (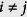), is determined as *a*_*ij*_ = *e*_*ij*_*A*_*ij*_ in a mutualistic interaction, *a*_*ij*_ = −*A*_*ij*_ in a competitive interaction, *a*_*ij*_ = −*g*_*ij*_*A*_*ij*_ and *a*_*ji*_ = −*a*_*ij*_/*g*_*ij*_ in an antagonistic interaction between the exploiter *i* and the resource *j*, *a*_*ij*_ = 0 and *a*_*ji*_ = −*A*_*ji*_ in amensalism, and *a*_*ij*_ = *c*_*ij*_*A*_*ij*_ and *a*_*ji*_ = 0 in commensalism. In these equations, *A*_*ij*_ is the encounter rate for interaction partners, *e*_*ij*_, *g*_*ij*_, and *c*_*ij*_ are conversion efficiencies when species *i* utilizes species *j* in a mutualistic, antagonistic, and commensalistic interaction, respectively. Although this biological assumption on interaction asymmetry was often not considered in community dynamics researches^27^,^32^, it is known to completely change a key prediction^33^. *s*_*i*_ is defined as *s*_*i*_ = *sδ*_*i*_, where *s* controls the magnitude of self-regulations and *δ*_*i*_ is potential self-regulation. Parameters *δ*_*i*_, *e*_*ij*_, *g*_*ij*_, *c*_*ij*_, *A*_*ij*_, and *X*_i_* are the absolute values randomly chosen from a normal distribution n(0, *σ*^2^) with mean 0 and standard deviation *σ* (Figures in the supporting information describe the case used in a uniform distribution). The intrinsic rate of change, *r*_*i*_, is determined to hold *dX*_*i*_/*dt* = 0 after imposing an equilibrium density for each species, *X*_i_*.

Stability analysis was based on a Jacobian community matrix. The consequences of a small perturbation in the population dynamics equilibrium model governed by equation 1 were considered. The dynamics of small deviations, *x*_*i*_, away from the equilibrium point, *X*_i_^*^, is given by 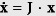 where 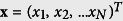, and *J* is the Jacobian matrix. The diagonal elements of *J* are represented by −*s*_*i*_*X*_i_^*^ and off-diagonal elements by *a*_*ij*_*X*_i_^*^. Stability was defined as the probability of local equilibrium stability, which was estimated as the frequency of locally stable systems across 1000 sample communities^34^. Direct calculation of the dominant eigenvalue also supports the results in the main text (Figs S7–S9). In addition, I directly calculated the Jacobian Matrix following May’s approach^27^,^32^, because some mathematical analysis assumes this approach (SI text). However, this assumption does not affect the present results (Figs S10 and S11).

Consider a community where *N* species may interact with each other with a probability *C* (connectance) through reciprocal interactions (antagonism, competition, or mutualism) and through unilateral interactions (amensalism or commensalism). In communities with only reciprocal interactions, proportions of antagonistic, mutualistic, and competitive interactions are defined as *p*_*a*_, *p*_*m*_, and *p*_*c*_ (= 1 − *p*_*a*_ − *p*_*m*_), respectively. In communities with unilateral interactions, proportions of commensalistic and amensalistic interactions are defined as *p*_*Co*_ and *p*_*Am*_ (=1 − *p*_*Co*_), respectively. The proportion of unilateral interactions within a community is defined as *p*_u_ (i.e., the proportion of reciprocal interactions is 1 − *p*_u_). The population dynamics of *N* species and the effect of unilateral interactions on population dynamics stability were evaluated by systematically changing *p*_u_.

The asymmetry of interaction strengths in communities with a single interaction type is controlled by changing the relative strengths of one interaction coefficient of all interacting pairs (Fig. 1). In antagonistic community, the relative strengths of interaction coefficients of victims, exploiters or half of victims and exploiters are changed by multiplying the asymmetry parameter *f* (0 < *f* < 1) by one interaction coefficient in each interaction pair. The same manner is applied to mutualistic and competitive communities.

## Additional Information

**How to cite this article**: Mougi, A. The roles of amensalistic and commensalistic interactions in large ecological network stability. *Sci. Rep.* **6**, 29929; doi: 10.1038/srep29929 (2016).

## Supplementary Material

Supplementary Information
