# Supplementary material for: The roles of amensalistic and commensalistic interactions in large ecological network stability
Source: Sci Rep. 2016 Jul 13;6:29929. doi: 10.1038/srep29929 (PMC4942820; doi:10.1038/srep29929)

Supplementary Information

The roles of amensalistic and commensalistic interactions in large ecological network stability

A. Mougi

Mathematical local stability analysis

Previous studies have proved the stability criteria of random community matrix comprised of each interaction type (predator-prey, mutualism, and competition) and all interaction types [1, 2]

Communities with a single interaction type

Community matrix, *J*, is a linear dynamic equation at an equilibrium point with elements. The system is locally stable if all *J* eigenvalues have negative real parts. Given the distribution assumptions from which interaction strengths are drawn [J_{ij} takes the value of a random variable *X* with mean $E(X) = 0$ and variance $V(X) = \sigma^2$], for large *N*, the stability conditions of random communities with predator-prey, mutualism, or competitive interactions follow Allesina and Tang (2012), respectively [1]:

$$\sigma\sqrt{NC}\left(1-\frac{E^2(|X|)}{\sigma^2}\right) < d\,, \qquad \text{(S1-a)}$$

$$(N-1)CE(|X|) < d\,, \qquad \text{(S1-b)}$$

$$\frac{\sqrt{NC}\left\{\sigma+\dfrac{(1-2C)E^2(|X|)}{\sigma}\right\}}{\sqrt{1-\dfrac{CE^2(|X|)}{\sigma^2}}} + CE(|X|) < d\,, \qquad \text{(S1-c)}$$

where *d* is the mean of self-regulations s_i.

Extreme interactions where one sign is close to zero (nearly unilateral interaction) in each interaction community were examined. In predator-prey communities the correlation of pairwise interactions $E(J_{ij}J_{ji})$ ($i \neq j$) becomes zero and $C = C/2$. The stability condition then changes to:

$$\sigma\sqrt{\frac{NC}{2}} < d\,. \qquad \text{(S2)}$$

This indicates that asymmetry of interaction strengths in predator-prey communities [(+ 0) or (−0)] destabilizes the system, because the stability condition is more restrictive in (S2) than in (S1-a). For example, assuming that *X* follows a normal distribution $n(0, \sigma^2)$, then $E^2(|X|)/\sigma^2 = \frac{2}{\pi}$. In this case, the left-hand sides of (S1-a) and (S2) are about $0.36\sigma\sqrt{NC}$ and $0.71\sigma\sqrt{NC}$, respectively.

In a similar way, when one interaction sign is close to zero in a mutualistic or competitive community, by using $E(J_{ij}J_{ji}) = 0$ and $C = C/2$ we reach stable conditions in each community.

$$\frac{(N-1)CE(|X|)}{2} < d \, , \tag{S3-a}$$

$$\sqrt{\frac{NC}{2}\left(\sigma^2 - \frac{CE^2(|X|)}{2}\right)} + \frac{C}{2}E(|X|) < d \, , \tag{S3-b}$$

The condition (S3-a) clearly indicates that asymmetry of interaction strengths in mutualistic communities stabilizes the system, because the stability condition is wider in (S3-a) than in (S1-b). Because of the complexities of stability conditions in competitive communities, it was specifically assumed that X follows a normal distribution $n(0, \sigma^2)$, then $E(|X|) = \sigma\sqrt{\frac{2}{\pi}}$ and $E^2(|X|)/\sigma^2 = \frac{2}{\pi}$. In this case, the stability conditions of competitive communities without or with asymmetrical interaction signs are respectively given by the following inequalities (note that the second terms in the left-hand side of inequalities are negligible for the larger N):

$$\sqrt{NC\left(1-\frac{2C}{\pi}\right)}\left(1+\frac{2(1-C)}{\pi-2C}\right) < \frac{d}{\sigma}, \tag{S4-a}$$

$$\sqrt{\frac{NC}{2}\left(1-\frac{C}{\pi}\right)} < \frac{d}{\sigma}, \tag{S4-b}$$

This indicates that asymmetry of interaction strengths in competitive communities stabilizes the system, because the stability condition is wider in (S4-b) than in (S4-a). These results support the numerical simulations in the main text (Fig 1).

Communities with unilateral interactions

Consider the systems with only unilateral interactions ($p_u = 1$). For randomly connected community with $J_{ii} = -d = (-s_i X_i^*)$, $E(J_{ij}) = 0$ and $CN >> 1$, the stability condition is given by:

$$\sqrt{N \cdot Var(J_{ij})}\left\{1 + \frac{E(J_{ij}J_{ji})}{Var(J_{ij})}\right\} < d \tag{S5}$$

[1]. In applying this stability condition to the present model, I assumed a random network with sufficient complexity ($CN >> 1$). Parameters and species abundance are set constant ($c_{ij} = c$, $A_{ij} = a$, $X_i^* = X^*$ and $s_i = s$). I further assumed that it holds that $c = p_{Co}(1 - p_{Co})$ so that $E(J_{ij}) = 0$. The diagonal elements are given by $J_{ii} = -sX^*$. Given that p_{Co} is not too close to one or zero, the off-diagonal elements are:

$$J_{ij} = caX^*, \quad \text{(S6-a)}$$

$$J_{ij} = 0, \quad \text{(S6-b)}$$

$$J_{ij} = -aX^*, \quad \text{(S6-c)}$$

$$J_{ij} = 0 \quad \text{(S6-d)}$$

for commensalist i and the comensalist's partner j and amensalist i and amensalist's partner j, occurring with probabilities, $p_{Co}C/2$, $p_{Co}C/2$, $(1 - p_{Co})C/2$ and $(1 - p_{Co})C/2$, respectively. Thus $Var(J_{ij})$ and $E(J_{ij} J_{ji})$ are calculated as

$$Var(J_{ij}) = X^{*2}p_{Co}C/2 + c^2X^{*2}(1 - p_{Co})C/2 \quad \text{(S7-a)}$$

and

$$E(J_{ij} J_{ji}) = 0, \quad \text{(S7-b)}$$

respectively. Substituting Eqns. (S6) and (S7) to Eqn, (S5), I have the stability condition for hybrid communities with unilateral interactions as:

$$\sqrt{p_{Co}\left(1+\frac{p_{Co}}{1-p_{Co}}\right)} < \frac{sX^*\sqrt{2}}{a\sqrt{NC}} \quad \text{(S8)}$$

This suggests that increasing the proportion of commensalistic interaction within a community monotonically increase the stability. This extreme analysis supports only case where parameter variations are extremely small (Fig. 2).

Communities with all interaction types

By extending the analysis of Allesina and Tang, [1] one can derive a stability criteria for communities comprised of all interaction types [2]. Given certain assumptions, the stability criteria can be defined as:

$$\text{Max}(r_e, r_s) < d, \quad \text{(S9)}$$

where

$$r_e = \sqrt{NC\left\{\sigma^2\left(1-\frac{p_{Co}+p_{Am}}{2}\right)-CE(|X|)^2\left(p_m-p_c+\frac{p_{Co}-p_{Am}}{2}\right)^2\right\}\cdot\left[1+\frac{E(|X|)^2\left\{(2p_m+2p_c+p_{Co}+p_{Am}-1)-C(p_m-p_c+0.5p_{Co}-0.5p_{Am})^2\right\}}{\sigma^2(1-0.5p_{Co}-0.5p_{Am})-CE(|X|)^2(p_m-p_c+0.5p_{Co}-0.5p_{Am})^2}\right]} \tag{S10-a}$$

and

$$r_s = (N-1)C\left(p_m-p_c+\frac{p_{Co}-p_{Am}}{2}\right)E(|X|). \tag{S10-b}$$

Noe that p_a, p_m, p_c, p_{Co}, and p_{Am} are the proportion of antagonistic, mutualistic, competitive, commensalistic, and amensalistic interactions within a community, respectively.

To compare the stability of communities with only reciprocal interactions and only unilateral interactions, two extremes are considered, communities with only reciprocal interactions $p_{\mathrm{u}} \approx 0$ and those with only unilateral interactions $p_u \approx 1$ as well as communities with perfectly balanced interaction types ($p_a= p_m= p_c$ and $p_{Co} = p_{Am}$). In both cases, $r_s = 0$ although r_e is different in each extreme ($p_{\mathrm{u}} \approx 0$ and $p_{\mathrm{u}} \approx 1$):

$$r_e = \sigma\sqrt{NC}\cdot\left(1+\frac{E(|X|)^2}{3\sigma^2}\right), \tag{S11-a}$$

$$r_e = \sigma\sqrt{\frac{NC}{2}}. \tag{S11-b}$$

This clearly indicates that communities with unilateral interactions are more stable than those with reciprocal interactions. It was also confirmed that communities with an unbalanced proportion of interaction types showed same tendency (see Fig 3 in the main text).

References

[1] Allesina S, Tang S. Stability criteria for complex ecosystems. Nature. 2012; 483:205–208.

[2] Coyte KZ, Schluter J, Foster KR. The ecology of the microbiome: Networks, competition, and stability. Science. 2015; 350:663–666.

Supplemental Figures

Fig. S1. Stability of communities with unilateral interactions (p_u = 1) with varying proportions of commensalism p_{Co}. (a) Effects of C, N was assumed to = 50. (b) Effects of N, C was assumed to = 0.2. Parameter values are s = 4.0 and σ= 0.5.

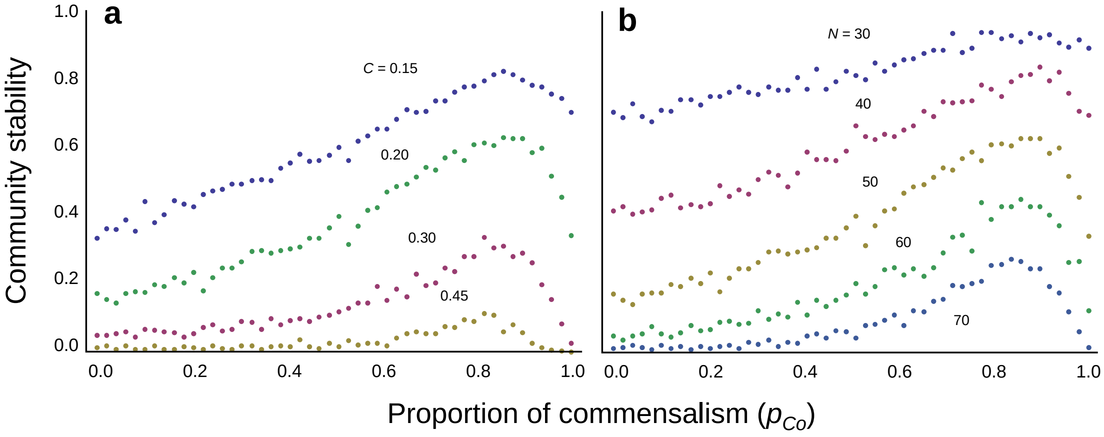

Fig. S2. Stability of communities with unilateral interactions ($p_u = 1$) with varying proportions of commensalism p_{Co}. (a) Effects of C, N was assumed to = 50. (b) Effects of N, C was assumed to = 0.2. Parameter values were randomly chosen from a uniform distribution (0 to 1) and s = 4.0.

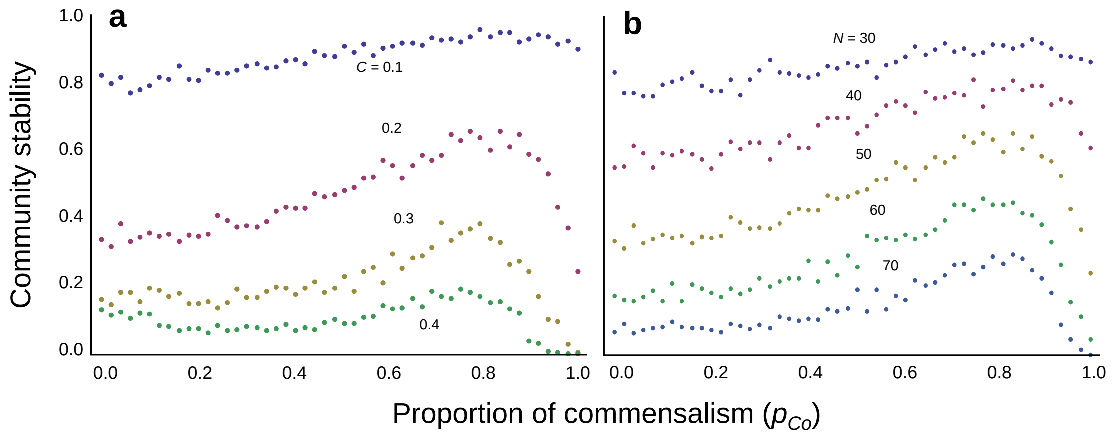

Fig. S3. Stability of hybrid communities with reciprocal and unilateral interactions with varying proportions of the unilateral interaction p_u. (a) Effects of C, N was assumed to = 50. (b) Effects of N, C was assumed to = 0.2. Parameter values are s = 4.0 and σ = 0.5.

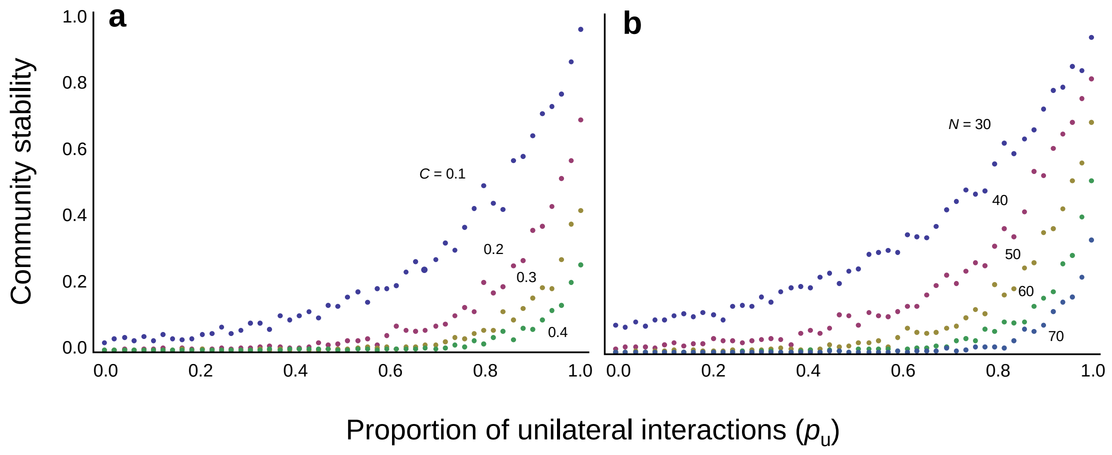

Fig. S4. Stability of hybrid communities with reciprocal and unilateral interactions with varying proportions of the unilateral interaction p_u. (a) Effects of C, N was assumed to = 50. (b) Effects of N, C was assumed to = 0.2. Parameter values were randomly chosen from a uniform distribution (0 to 1) and s = 4.0.

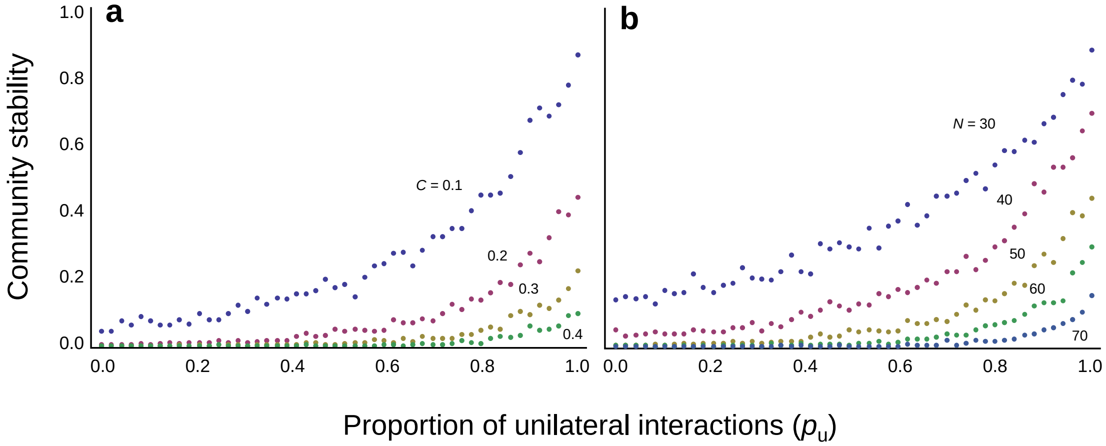

Fig. S5. Statistical quantities of off-diagonal elements of Jacobian matrix varying proportion of unilateral interactions. It was assumed that $p_a = p_m = p_c$, and $p_{Co} = p_{Am}$. Parameter values are N = 50, C = 0.2, s = 4.0, and σ = 0.3. Dotted lines indicate zero. The absolute values of correlation are extremely small ($< 10^{-5}$).

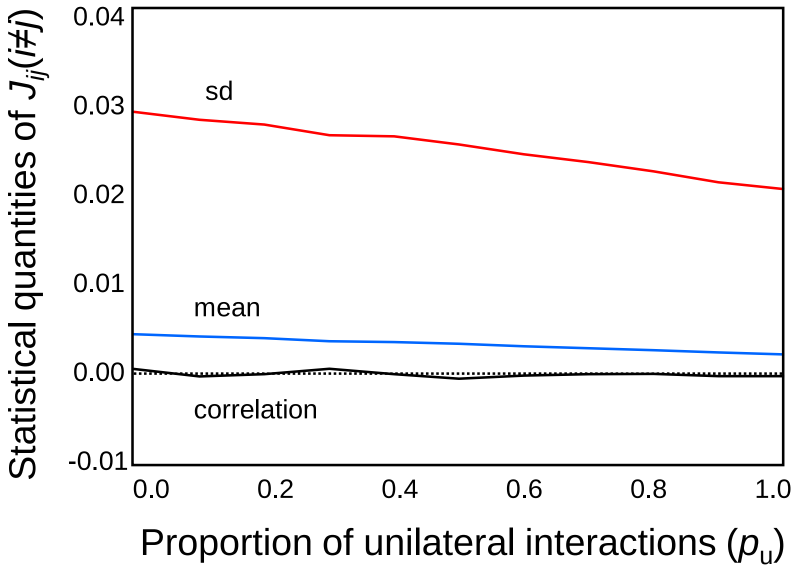

Fig. S6. Statistical quantities of off-diagonal elements of Jacobian matrix and stability of communities comprised of only reciprocal interactions (p_u =0) with varying reduction rate of σ and magnitudes of J_{ij} ($i \neq j$). In (a-b) and (c-d), statistical quantities are changed without changing proportion of unilateral interactions (p_u =0), by reducing the absolute values of σ and J_{ij} ($i \neq j$), respectively. Red and blue dashed lines indicate values of standard deviation and absolute mean of J_{ij} ($i \neq j$) in communities comprised of only unilateral interactions (p_u = 1.0 in Fig. S5). Black solid lines in (b) and (d) indicate community stability in communities comprised of only reciprocal interactions (p_u =0). Black dashed lines indicate community stability in communities comprised of only unilateral interactions (p_u = 1.0 in Fig. S5). Black circles indicates the points where each quantities in non-asymmetrical and perfect asymmetrical communities are same. The magnitudes of statistical quantities shown in communities with only unilateral interactions (black point in (c)) are not enough large to explain the stability (black point in (d)). The magnitudes of σ do not affect to stability.

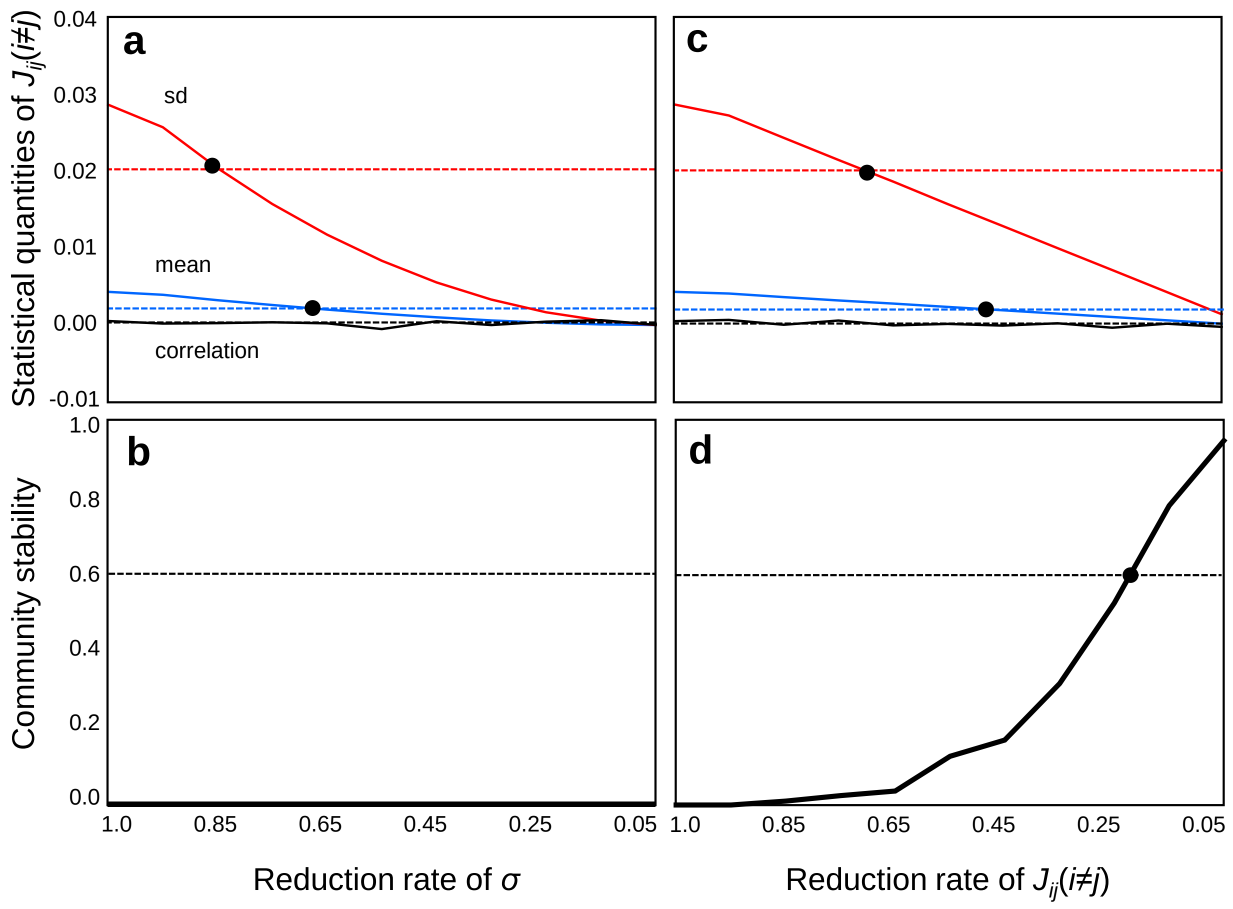

Fig. S7. Mean dominant eigenvalue with varying asymmetry of interaction strengths *f*. Dotted line in panel (a) indicates zero line. Other information is same as Fig. 1.

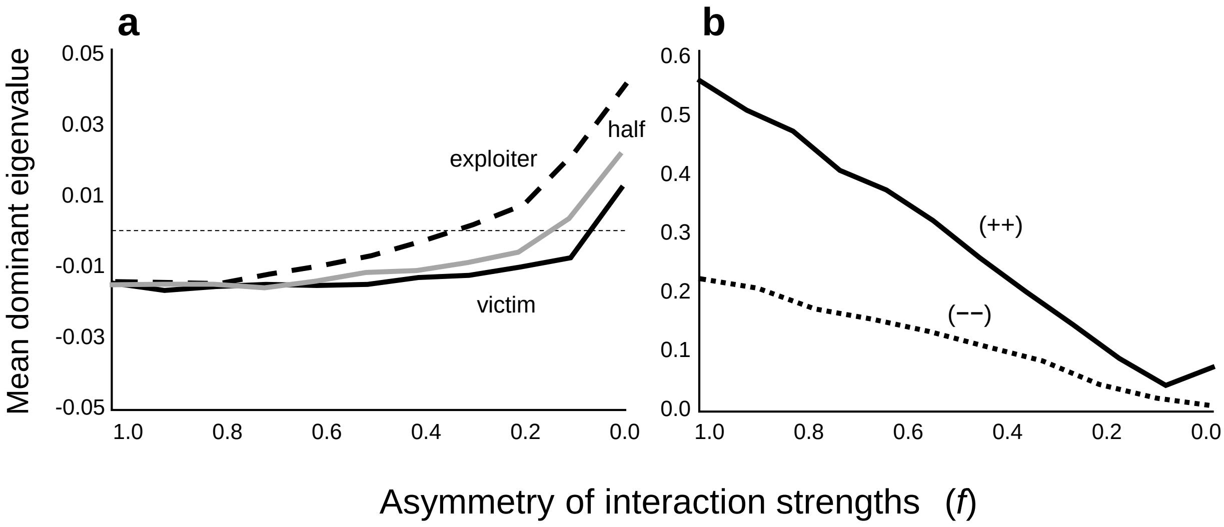

Fig. S8. Mean dominant eigenvalue with varying with varying proportion of commensalism p_{Co}. Dotted line in panel (a) indicates zero line. Other information is same as Fig. 2.

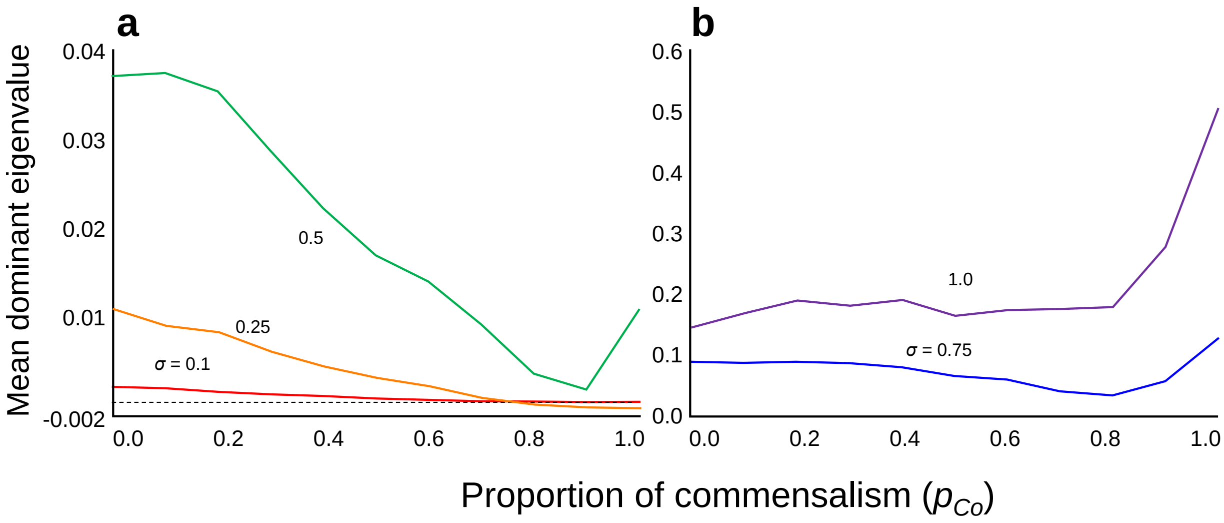

Fig. S9. Mean dominant eigenvalue with varying with varying variable proportions of unilateral interactions p_u. Dotted line in panel (b) indicates zero line. Other information is same as Fig. 3.

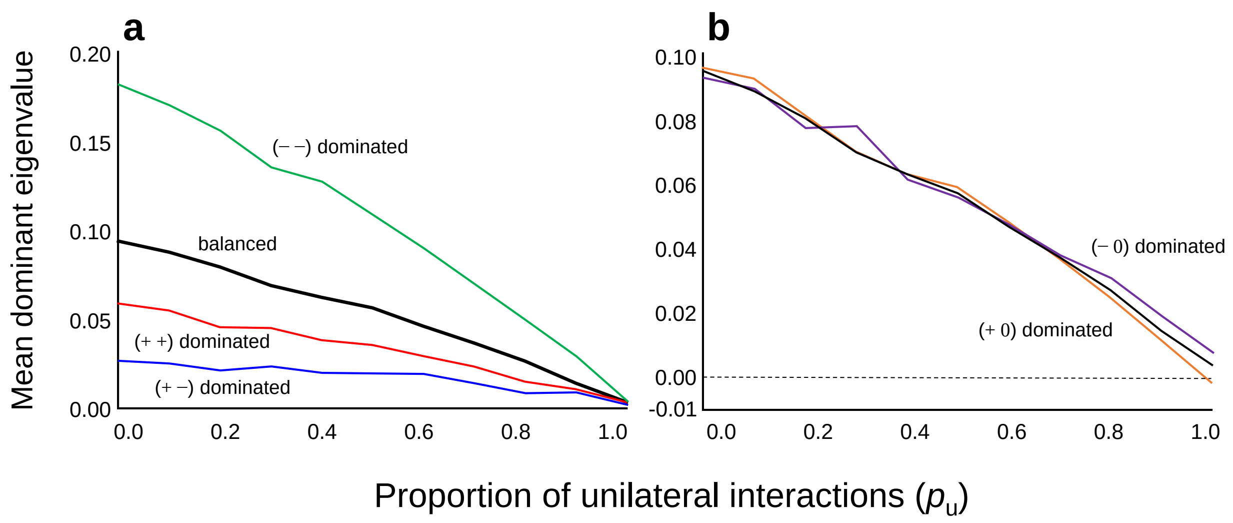

Fig. S10. Effects of interaction strength asymmetry on stability of communities with reciprocal interactions (p_u = 0). (a) Antagonistic community, (b) Competitive or mutualistic community. In the simulations, Jacobian Matrix is directly calculated following May's approach (Methods). Parameter values are N = 50, C = 0.2, s_i = 1.0, and σ = 0.5.

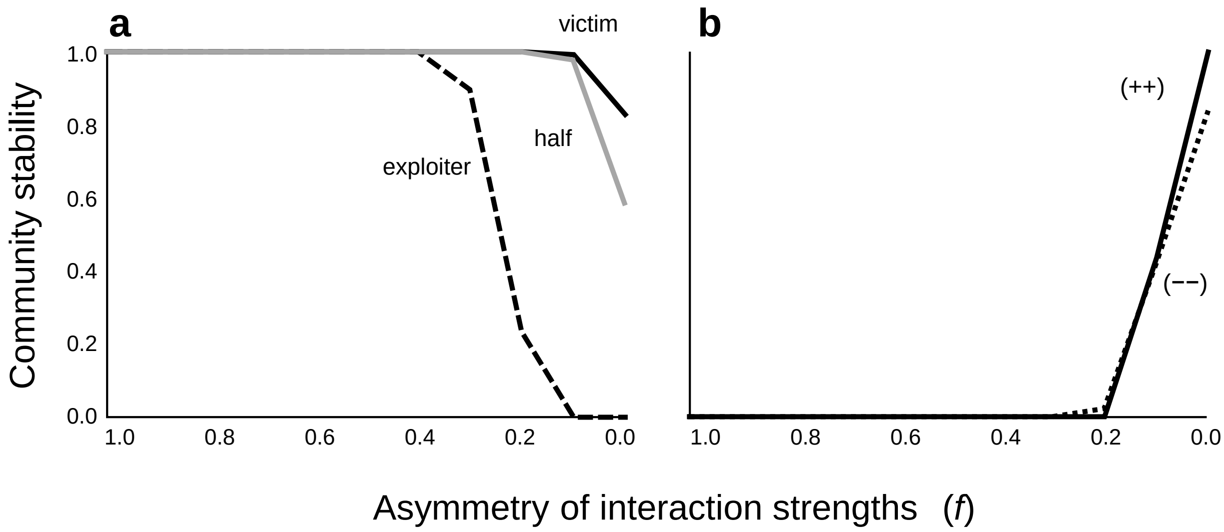

Fig. S11. Stability of hybrid communities with reciprocal and unilateral interactions with variable proportions of unilateral interactions p_u. (a) Effects of different community composition of the reciprocal interactions, balanced interactions, p_a dominated, p_m dominated, and p_c dominated. Black, blue, red, and green dots indicate different proportions of reciprocal interactions, (p_a, p_m, p_c) = (1/3, 1/3, 1/3), (0.6, 0.2, 0.2), (0.2, 0.6, 0.2), and (0.2, 0.2, 0.6), respectively. (b) Effects of different community composition of the unilateral interactions, balanced interactions, p_{Am} dominated, and p_{Co} dominated. Black, purple, and orange dots indicate different unilateral interaction proportions, p_{Co} = 0.5, 0.2, and 0.8, respectively. It was assumed that $p_a = p_m = p_c$. In the simulations, Jacobian Matrix is directly calculated following May's approach (Methods). Parameter values are N = 50, C = 0.2, s_i = 1.0, and σ = 0.5.

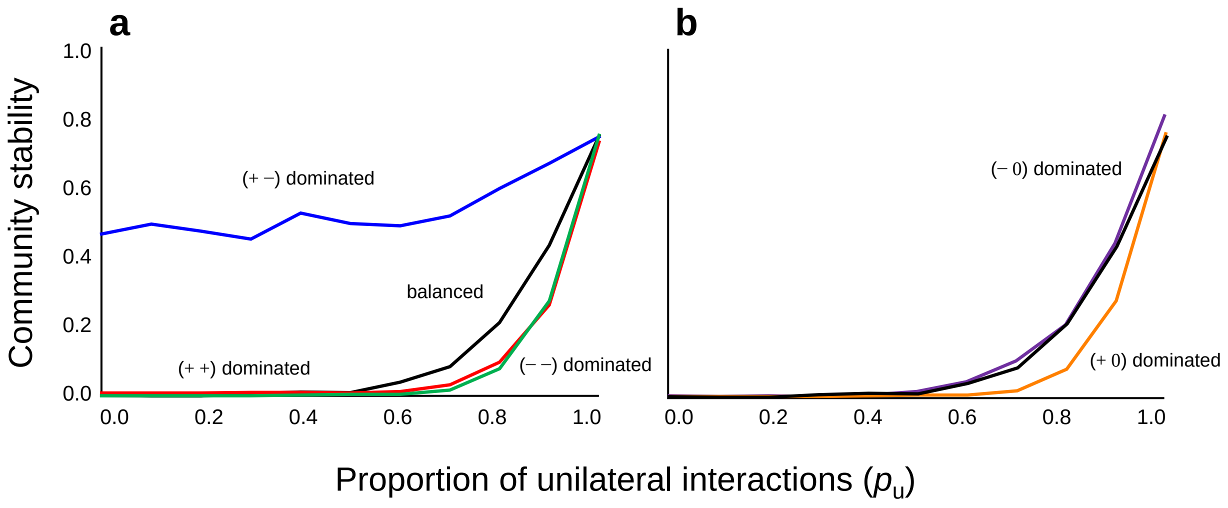

Supplement: Supplementary Information [file srep29929-s1.pdf]
